# A Comparison of Bioimpedance Analysis vs. Dual X-ray Absorptiometry for Body Composition Assessment in Postpartum Women and Non-Postpartum Controls

**DOI:** 10.3390/ijerph192013636

**Published:** 2022-10-20

**Authors:** Valene Garr Barry, Samantha L. Martin, Paula Chandler-Laney, Ebony B. Carter, Camille S. Worthington

**Affiliations:** 1 Department of Obstetrics and Gynecology, Washington University School of Medicine in St. Louis, St. Louis, MO 63110, USA; 2 Department of Obstetrics and Gynecology, The University of Alabama at Birmingham, Birmingham, AL 35233, USA; 3 Department of Nutrition Sciences, The University of Alabama at Birmingham, Birmingham, AL 35294, USA

**Keywords:** bioelectrical impedance analysis, postpartum, dual-X-ray absorptiometry, women, body composition, fat mass, fat-free mass

## Abstract

Postpartum fat mass (FM) and fat-free mass (FFM) may be informative predictors of future disease risk among women; hence, there is growing use of bioelectrical impedance analysis (BIA) to quantify FFM and FM among postpartum women due to the quick, non-invasive, and inexpensive nature of BIA. Despite this, very few studies have examined BIA’s performance, and it remains unclear as to whether specific BIA equations are needed for postpartum women. To explore these questions, we measured total body FFM and FM with a multi-frequency, segmental BIA, and dual-X-ray absorptiometry (DXA) in (1) women at one and four months postpartum (*n* = 21); and (2) height- and weight-matched non-postpartum women (controls, *n* = 21). BIA was compared to DXA using Deming regression models, paired *t*-tests, and Bland–Altman plots. Between-group comparisons were performed using an analysis of variance models. The mean difference between DXA and BIA was 1.2 ± 1.7 kg FFM (*p* < 0.01) and −1.0 ± 1.7 kg FM (*p* < 0.05) in postpartum women at both time points. The measurements of longitudinal changes in FFM and FM were not significantly different between BIA and DXA. Furthermore, there was no significant difference in BIA’s performance in postpartum vs. non-postpartum women (*p* = 0.29), which suggests that population-specific equations are not needed for postpartum women. The results of this study suggest that BIA is a suitable method to assess postpartum body composition among women at one and four months postpartum, using existing age-, race-, and sex-adjusted equations.

## 1. Introduction

The postpartum period, up to one year following delivery, has been highlighted as a tipping point for a woman’s future health [1,2,3,4]. In particular, postpartum weight retention, which affects up to 75% of postpartum women, has been underscored as a significant contributor to future obesity and chronic disease risk [5,6,7]. There is growing interest in postpartum body composition due to evidence that postpartum fat mass (FM) and fat-free mass (FFM) may be more informative predictors of future disease risk than body weight or body mass index (BMI) alone [1,8]. As a result, comprehensive assessments of body composition that can aid in understanding the composition, distribution, and trajectory of postpartum changes in FM, FFM, and weight are needed to support the further investigation of postpartum health.

Bioelectrical impedance analysis (BIA) is often used to assess postpartum body composition in clinical and research settings because it is more affordable, accessible, and less burdensome than other methods, including magnetic resonance imaging (MRI), computed tomography (CT), and dual-X-ray absorptiometry (DXA) [9]. BIA estimates body composition by measuring the body’s resistance to an applied current and employing population- and device-specific prediction equations [9]. Historically, BIA has been considered less accurate than other methods, especially under non-standard conditions, including certain disease states, asymmetrical body shapes, and altered hydration states [9,10,11]; however, advancements in BIA devices, such as the implementation of multi-frequency and segmental approaches, have been shown to improve BIA’s performance and robustness against deviations from the norm, such as those which occur during the postpartum period [9,12,13]. Nevertheless, there is a paucity of evidence to either support or refute the use of BIA to measure postpartum body composition.

BIA measures postpartum body composition using age-, sex-, and race-adjusted equations that have been developed using non-postpartum women. Such equations do not account for the altered physiology of the postpartum body, which includes (1) variations in body shape (e.g., larger waist circumference) and (2) dramatic perturbations of hydration and body water from pregnancy and lactation/breastfeeding [7]; however, there are no studies that have compared the performance of BIA equations, using postpartum and non-postpartum women, to determine whether the new equations are needed. Very few studies have compared BIA with a reference method, such as DXA, in postpartum women. Furthermore, most comparison studies in postpartum women have (1) only evaluated FM and (2) used BIA devices that are no longer commercially available, which due to advancements in design and functionality, limits their relevance when making decisions regarding the use of newer and potentially improved BIA devices [14,15,16].

The purpose of this study was to examine the performance of a multi-frequency, segmental BIA, which provides a longitudinal assessment of body composition in postpartum women; hence, we compared estimates of FFM and FM between BIA and DXA in two groups of women: (1) women at one and four months postpartum; and (2) height- and weight-matched, weight-stable, non-postpartum women.

## 2. Methods

### 2.1. Study Design and Participants

In this study, we performed a secondary analysis of data from 21 postpartum women who participated in a longitudinal cohort study of maternal factors that influence breast milk composition between January 2017 and March 2018. We selected 21 non-postpartum women, who were matched one to one with the postpartum women by height and weight, from a group of 72 healthy, weight-stable women who had not given birth in the prior year, and who had participated in a study of body composition assessment methods between October 2019 and March 2020. Both studies recruited participants from the University of Alabama at Birmingham (UAB) and the surrounding areas. For postpartum women, the inclusion criteria were age ≥19 years old, 1 month postpartum, and exclusively breastfeeding (i.e., no formula supplementation). The exclusion criteria for postpartum women were smoking, illicit drug use, diagnosis, history, or medication use that would influence metabolism (e.g., type 1 or 2 diabetes, thyroid disorders, polycystic ovary syndrome), and delivery of an infant with medical conditions that would interfere with adequate feeding or development (e.g., failure to thrive, cleft lip or palate, dysphagia, feeding/swallowing conditions requiring a feeding tube). For the non-postpartum control group, the inclusion criteria were age ≥18 years old with a BMI of 18.45–45 kg/m^2^. Exclusion criteria for the control group were childbirth within the last year, weight fluctuations ±4.54 kg in the prior six months; previous diagnoses of chronic or critical diseases, such as cancer, cardiovascular, or cerebrovascular events; and use of potassium supplements, diuretics, or drugs that are known to regulate fluid balance. For both groups, those with contraindications for bioimpedance were excluded, including amputations, artificial joints, pins, plates, or other types of metal objects in the body; pacemakers or automatic defibrillators; coronary stents or metal suture material in the heart. The UAB Institutional Review Board approved the study, and all participants provided written and verbal consent.

### 2.2. Measurements and Tests

For both cohorts, all measurements and tests were performed at the UAB Human Physiology Core facilities using the same instruments and protocols. For postpartum women, body composition measurements were performed at one and four months postpartum. Measurements were taken immediately following a complete expression of milk from one breast. For non-postpartum women, body composition measurements were performed in the follicular phase (i.e., days 1–8) of the menstrual cycle. For both cohorts, study visits occurred between 8:00 am and 10:00 am following an overnight fast (i.e., no food or drink except plain water) of at least 10 h. Before testing, participants were asked to avoid drinking alcohol within 24 h of testing, to avoid exercise or sauna use within 12 h of testing, and to refrain from using hand or body lotion the morning of the testing.

Anthropometric measurements were assessed for all participants, including height, weight, and waist circumference. Height was measured to the nearest 1 mm using a Seca^®^ 264 digital stationary stadiometer (seca GmbH & Co. KG, Hamburg, Germany). Weight was measured to the nearest 45 g, with women wearing minimal clothing using the platform scale of the BIA. Waist circumference was measured to the nearest 0.1 cm at the umbilicus using a flexible measuring tape. The body composition measures of FM, FFM, TBW, intracellular water (ICW), and extracellular water (ECW) were collected using a multi-frequency segmental bioimpedance analyzer device (seca^®^ medical Body Composition Analyzer 515/514; seca GmbH & Co. KG, Hamburg, Germany). The seca mBCA 514 is an 8-point (e.g., lead) analyzer that uses contact-plate electrodes positioned on a standing platform and handrails for optimal use in clinical settings. Resistance and reactance were measured at 19 frequencies: 1, 1.5, 2, 3, 5, 7.5, 10, 15, 20, 30, 50, 75, 100, 150, 200, 300, 500, 750, and 1000 kHz. Body composition values were estimated using proprietary equations that account for age, sex, race/ethnicity, height, and weight. FM and FFM were also measured by DXA (iDXA, GE Healthcare Lunar, Madison, WI, USA). The DXA was phantom calibrated daily to assure the instrument’s reliability. Urine pregnancy tests were administered before each participant underwent a DXA scan. All participants self-reported their age and race/ethnicity.

### 2.3. Statistical Analysis

Postpartum women were pair matched (1:1) with healthy controls using their 1-month postpartum height (±3.0 cm) and weight (±5.0 kg). Characteristics of each group were calculated and compared between the two groups using a student’s t-test for continuous variables and a chi-squared test for categorical variables. Longitudinal changes in body composition between one and four months postpartum were examined using paired *t*-tests. FM and FFM measurements from BIA were compared with DXA for (1) one month postpartum women, (2) four month postpartum women, and (3) non-postpartum controls using the slope and intercept of Deming linear regression models. Deming regression models were used because they consider potential errors in both measurements, as neither BIA nor DXA is considered a gold standard. Bland–Altman plots were constructed from the average vs. the difference in FM and FFM from BIA and DXA. The slopes of the trendlines for the Bland–Altman plots were examined for evidence of systematic bias. FM and FFM values were also compared between BIA and DXA using paired *t*-tests, and the mean differences were compared between groups using an analysis of variance (ANOVA) model. All analyses were performed using SAS^®^ version 9.4 (SAS Institute, Cary, NC, USA), and the statistical significance was set at *p* < 0.05.

## 3. Results

Study participants (*n* = 42) are characterized by group in Table 1. Twenty-one postpartum women and 21 height- and weight-matched non-postpartum controls were included in the present analyses. The average difference between one-month postpartum women and matched non-postpartum controls was −0.2 ± 1.1 cm for height and 0.7 ± 4.1 kg for weight. Overall, participants were 30 ± 6 years old [range: 19–45 years], 86% White and 14% Black, with a BMI of 29.7 ± 7.6 kg/m^2^ (range: 18.5–44.5). There were no significant differences in demographic or body composition measures between the groups; however, the postpartum women were slightly older (*p* = 0.07), more racially homogeneous (95% vs. 76.2% White, *p* = 0.08), and had a slightly larger waist circumference at one month postpartum (100.1 ± 16.0 cm vs. 89.9 ± 19.1 cm, *p* = 0.07), compared with the non-postpartum controls.

The longitudinal changes in the body composition of postpartum women between one and four months postpartum are shown in Table 2. Eighteen (86%) of the 21 postpartum women attended the four-month postpartum study visit. Overall, postpartum women did not show significant changes in weight (−0.8 ± 2.9 kg; *p* = 0.23), FFM_DXA_ (−0.4 ± 1.0 kg; *p* = 0.09), or FM_DXA_ (−0.4 ± 2.4 kg; *p* = 0.45); however, there was a significant decrease in ECF between one and four months postpartum (*p* = 0.0021), which indicates the normalization of hydration and fluid volumes. The longitudinal changes in FFM (*p* = 0.41) and FM (*p* = 0.74) were not statistically different when measured with BIA vs. DXA.

**Figure 1:**
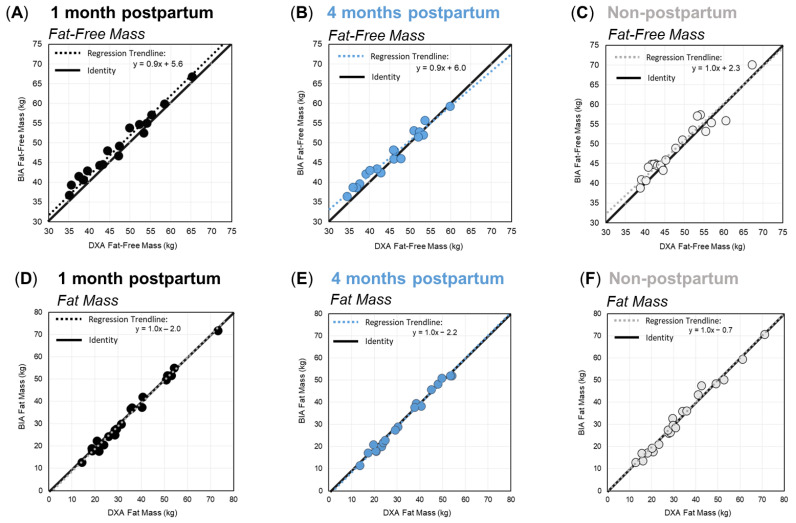
Deming regression plots for fat-free mass (**A**–**C**) and fat mass (**D**–**F**) for women at one month postpartum (black), four months postpartum (blue), and the non-postpartum control group (grey). Solid diagonal lines represent the line of identity, and dashed lines show the respective regression trendlines.

Deming regression analyses for FFM and FM, by group and time point, are shown in Figure 1. For FFM (Figure 1A–C), the slope of the regression lines showed a constant and proportional overestimation of FFM by BIA at one month postpartum. At four months postpartum, and among non-postpartum controls, BIA overestimated FFM at lower values, and the bias trend shifted towards underestimation at higher FFM values. For FM (Figure 1D–F), the slope of the regression line was 1.0, and it was aligned with the line of identity for each group, indicating no proportional bias between the two methods for the prediction of FM in any group.

**Figure 2:**
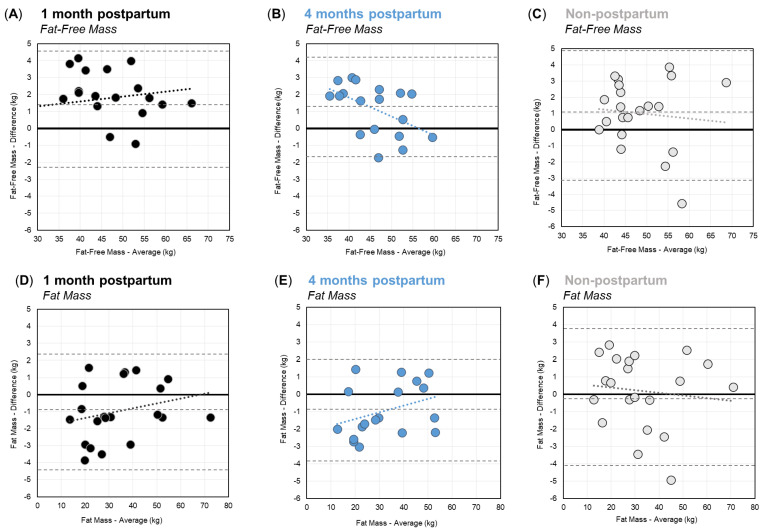
Bland–Altman Plots for Fat-Free Mass (**A**–**C**) and Fat Mass (**D**–**F**) for women at one month postpartum (black), four months postpartum (blue), and the non-postpartum control group (grey). Dashed lines show the respective mean differences and limits of agreement.

Bland–Altman plots showing the agreement between BIA and DXA for FFM and FM are shown in Figure 2. For FFM, Bland–Altman plots (Figure 2A–C) showed that the difference between BIA and DXA increased at higher FFM values at one month postpartum. Conversely, at four months postpartum, and for non-postpartum controls, the difference between BIA and DXA decreased at higher FFM values. The mean differences in FFM were 1.2 ± 1.7 kg, 1.2 ± 1.5 kg, and 1.0 ± 2.1 kg at one-month postpartum, four months postpartum, and among non-postpartum controls, respectively. Paired *t*-tests indicated that the FFM estimates from BIA were significantly higher than DXA for postpartum women at both time points (*p* < 0.01), and for non-postpartum controls (*p* < 0.05). There was no significant difference in the agreement (i.e., mean difference), of BIA with DXA, between groups (*p* = 0.29). The overall mean difference in FFM was 1.1 ± 1.9 kg (4.2%).

Second, for FM, the Bland–Altman plots (Figure 2D–F) showed that the bias between BIA and DXA shifted from underestimation to overestimation as the FM increased in postpartum women at both time points; however, for non-postpartum controls, BIA shifted from a slight overestimation at lower FM values to an underestimation at higher FM values. The mean difference between BIA and DXA for FM was −1.0 ± 1.7 kg, −1.0 ± 1.5 kg, and −0.2 ± 2.1 kg at one month postpartum, four months postpartum, and among non-postpartum controls, respectively (Table 3). Paired *t*-tests indicated that BIA and DXA estimates of FM were significantly different at one and four months postpartum (*p* < 0.05), but the BIA’s estimates of FM were not significantly different from DXA among non-postpartum controls (*p* = 0.67). There was no significant difference in the agreement (mean difference), of the BIA with DXA, between groups (*p* = 0.27). The overall mean difference in FM was −0.6 ± 1.9 kg (6.2%).

## 4. Discussion

The main finding of this study is that, compared with DXA, BIA overestimated FFM by 1.2 ± 1.7 kg (4.2 ± 2.6%) and underestimated FM by 1.0 ± 1.7 kg (6.4 ± 5.1%) in postpartum women, at both one and four months postpartum. Given that the bias between DXA and BIA was consistent at both time points, BIA’s measurement of longitudinal changes in FFM and FM was not statistically different from DXA. These findings suggest that BIA may be best suited for the assessment of relative changes in body composition among postpartum women rather than to assess absolute FM or FFM at any given time point. These findings are consistent with those of previous studies, which have found similar cross-sectional and longitudinal differences in FM between BIA and DXA in (1) lactating women who are overweight or obese at 2–3 months postpartum, and who underwent a 12-week weight loss intervention [14]; (2) lactating women at 2–3 months postpartum [17]; and (3) women at two weeks postpartum (not accounting for lactation/breastfeeding status) [16]. However, none of the previous studies reported comparisons for FFM, which is an important factor to consider for metabolic health, especially with weight loss, and the BIA instruments used in those studies are no longer commercially available. Therefore, the results of this study add a comparison between BIA and DXA to the literature, which focuses on postpartum women, and that includes the FFM and FM compartments using a contemporary BIA device.

The secondary finding of this study is that there was no significant difference in the performance of BIA’s standard equations for FM or FFM between postpartum women and non-postpartum controls. It should be noted that BIA’s estimates of FM were only significantly lower than DXA in postpartum women. It is likely that BIA’s FM estimates are only different from DXA in postpartum women because the equations used were designed for, and validated in, non-postpartum women, which suggests that BIA’s FM estimates might be slightly improved if there were specific FM equations for postpartum women. The need for such equations has been previously suggested by Medoua et al., who hypothesized that lactation was the primary reason for the observed bias in the BIA’s FM estimates among postpartum women [17]; however, to date, no studies have compared BIA’s performance between lactating and non-lactating postpartum women, therefore, in this study, we aimed to test this hypothesis by matching postpartum women who exclusively breastfed, with non-postpartum (i.e., non-lactating) controls. Although we did not find a significant difference between groups, the results of this study suggest that population-specific equations might improve FM estimates, but such equations are not necessary for FFM. Given that BIA equations tend to be population- and device-specific [10,18], those that use BIA in postpartum women might do well to standardize their specific device’s measurements against a suitable reference method, such as DXA or isotope dilution, in a sample that is similar to the target population.

This study should be interpreted in consideration of the following strengths and limitations. The primary strength of this study is that it examined the performance of a contemporary bioimpedance analyzer in postpartum women. We used the seca mBCA 514, which is a modern, eight-electrode, segmental multifrequency device with updated equations, that include both resistance and reactance, and it adjusts for differences in body shape, which likely improves measurement accuracy [9,12,13,19,20,21]. Previous studies have demonstrated that this device has highly reproducible and upright positioning, and it uses metal plates instead of gel electrodes, which makes it optimal for high-throughput use in clinical and research settings [19]. This study is also strengthened by the performance of assessments at multiple time points without intervention among postpartum women, and it is further strengthened by the inclusion of height- and weight-matched controls.

The potential limitations of this study include the small sample size and the racial homogeneity of the participants. In addition, we compared BIA with DXA, which is a rigorous method that was preferable to isotope dilution due to the participant’s active breastfeeding status, but DXA is not a gold standard. Moreover, this study performed the earliest measurements at one month postpartum, and thus, the period of rapid fluid changes that occur at 2–4 weeks postpartum had concluded [22,23,24]. Furthermore, this study did not include non-lactating postpartum women, who constitute slightly more than half of women at three months postpartum [25], and have significant differences in body composition compared with lactating postpartum women [8,26]. Lastly, the results of this study may not be generalizable among all BIA instruments given that BIA equations tend to be device-specific. Future studies should evaluate BIA’s performance with postpartum body composition in larger, more diverse cohorts that include both lactating and non-lactating women, as well as comparisons across devices.

Overall, this study suggests that, relative to DXA, BIA is a suitable method to assess postpartum body composition at one and four months postpartum, using the existing age-, race-, and sex-adjusted equations. BIA produced similar measurements in terms of longitudinal changes, which suggests that BIA may be most suitable for the clinical management of postpartum weight loss. Applications where the threshold for measurement accuracy may be higher, such as between-group comparisons and intervention studies, should consider the potential over- or underestimation of FFM and FM. Nevertheless, BIA is non-invasive, cost-effective, easily accessible, and often portable, which is especially advantageous in settings with limited access to diagnostic tools [9]. In general, the results of this study suggest that BIA is a valid tool to aid clinicians and researchers seeking to understand the composition and trajectory of postpartum weight change, which will be important given the potential for postpartum weight changes to impact women’s future health outcomes.

## Figures and Tables

**Table 1:** Participant characteristics by group (*n* = 42).

Characteristic	Postpartum Women (*n* = 21)	Non-Postpartum Women (*n* = 21)	*p*-Value
	Mean ± SD or *n* (%)	Mean ± SD or *n* (%)	
Age, years	31.5 ± 4.8	27.7 ± 9.8	0.07
Race Black White	1 (4.8) 20 (95.2)	5 (23.8) 16 (76.2)	0.08
Height, cm	164.8 ± 6.0	164.3 ± 5.8	0.69
Weight, kg	80.4 ± 23.0	80.4 ± 22.6	0.90
BMI, kg/m^2^	29.6 ± 7.5	29.8 ± 7.8	0.97
Obesity, BMI < 34 kg/m^2^ BMI ≥ 34 kg/m^2^	14 (67.7) 7 (33.3)	14 (67.7) 7 (33.3)	1.00
Waist Circ., cm	100.1 ± 16.0	89.8 ± 19.1	0.07
Fat-Free Mass, DXA kg	46.3 ± 8.0	47.8 ± 7.7	0.51
Fat Mass, DXA, kg	34.1 ± 15.1	32.7 ± 15.5	0.35
Total Body Water, BIA, kg	35.9 ± 5.8	35.6 ± 4.4	0.78
Extracellular Water, kg	15.7 ± 3.1	15.8 ± 2.3	0.84
Intracellular Water, kg	20.3 ± 2.9	19.9 ± 2.3	0.53

Characteristics are shown for postpartum women at one-month postpartum. *p*-values are for between-group comparisons using the *t*-test or chi-squared test. Abbreviations: BMI, body mass index; BIA, bioelectrical impedance analysis; DXA, dual X-ray absorptiometry.

**Table 2:** Change in body composition between one and four months postpartum.

Characteristic	1 Month Postpartum (*n* = 18)	4 Months Postpartum (*n* = 18)	Change	*p*-Value
	Mean ± SD	Mean ± SD	Mean ± SD	
Weight, kg	78.9 ± 18.7	78.1 ± 19.7	−0.8 ± 2.8	0.23
BMI, kg/m^2^	29.4 ± 6.9	29.1 ± 7.2	−0.3 ± 1.0	0.19
Waist Circ., cm	98.9 ± 12.5	99.0 ± 14.0	−0.1 ± 3.9	0.96
Fat-Free Mass, DXA, kg	45.8 ± 7.1	45.4 ± 7.3	−0.4 ± 1.0	0.09
Fat Mass, DXA, kg	33.2 ± 12.7	32.7 ± 13.3	−0.4 ± 2.4	0.45
Total Body Water, BIA, L	35.0 ± 4.8	34.4 ± 5.2	−0.6 ± 1.2	0.05
Extracellular Water, L	15.5 ± 2.4	15.1 ± 2.4	−0.4 ± 0.5	**0.0021**
Intracellular Water, L	19.5 ± 2.5	19.5 ± 2.8	0.0 ± 0.5	0.76

*p*-values test the significance of longitudinal changes by paired *t*-test. Bold values indicate *p* < 0.05. Participants with missing 4-month values were omitted from analyses (*n* = 3). Abbreviations: BMI, body mass index; BIA, bioelectrical impedance analysis; DXA, dual X-ray absorptiometry.

**Table 3:** Paired *t*-tests of Mean Difference for Fat-Free Mass and Fat Mass.

Component	Parameter	DXA	BIA	Mean Difference ^†^	*p*-Value
		Mean ± SD	Mean ± SD	Mean ± SD	
Fat-Free Mass, kg	One month Postpartum	46.3 ± 8.0	47.5 ± 7.3	1.2 ± 1.7 **	0.29
	Four months Postpartum	45.4 ± 7.3	46.5 ± 6.5	1.2 ± 1.5 **	
	Non-postpartum controls	47.8 ± 7.7	48.8 ± 7.5	1.0 ± 2.1 *	
Fat Mass, kg	One month postpartum	34.1 ± 15.1	33.2 ± 15.6	−1.0 ± 1.7 *	0.27
	Four months postpartum	32.7 ± 13.3	31.8 ± 13.8	−1.0 ± 1.5 *	
	Non-postpartum controls	32.7 ± 15.5	32.5 ± 15.8	−0.2 ± 2.1	

† Mean difference = BIA–DXA; Paired *t*-test for DXA vs. BIA: * *p* < 0.05; ** *p* < 0.01. *p*-values are for a comparison of the mean differences across groups by analysis of variance. Abbreviations: BIA, bioelectrical impedance analysis; DXA, dual X-ray absorptiometry; pp, postpartum.

## Data Availability

Please contact the corresponding author (valene@wustl.edu) for inquiries regarding data availability.
